# Effects of lactobacillus and serratopeptidase supplementation on growth performance, hematology, immune response, and intestinal morphology in broiler chicks

**DOI:** 10.1016/j.psj.2025.105500

**Published:** 2025-06-27

**Authors:** Muhammad Mushtaq, Hanan Al-Khalaifah, Qurat ul-Ain, Imran Ullah, Muqadar shah, Ibrahim A. Alhidary, Shabana Naz, Rifat Ullah Khan, Ala Abudabos

**Affiliations:** a Department of Poultry Science, Faculty of Animal Husbandry and Veterinary Sciences, The University of Agriculture, Peshawar, Pakistan; b Environment and Life Sciences Research Center, Kuwait Institute for Scientific Research, Safat, Kuwait; c College of Veterinary Sciences, Faculty of Animal Husbandry and Veterinary Sciences, The University of Agriculture, Peshawar, Pakistan; d Department of Zoology, Government College University, Faisalabad, Pakistan; e Department of Agriculture, School of Agriculture and Applied Sciences, Alcorn State University, 1000 ASU Drive, Lorman, Mississippi 39096-7500, USA; f Department of Animal Production, College of Food and Agriculture Science, King Saud University, Riyadh, Saudi Arabia

**Keywords:** Broilers, Serratopeptidase, *Lactobacillus*, Histomorphology

## Abstract

This study evaluated the effects of different levels of *Lactobacillus* and serratopeptidase enzyme supplementation on growth performance, immune response, hematological parameters, and intestinal histomorphology in broiler chicks. A total of 320-day-old male Hubbard broilers were assigned to four dietary treatments (five replicates) in a completely randomized design as follow: a control diet, and diets supplemented with *Lactobacillus* at 2 × 10¹°CFU/kg, 3 × 10¹°CFU/kg, and 4 × 10¹°CFU/kg, each combined with 5 mg/kg serratopeptidase. Feed intake, weight gain, and feed conversion ratio (FCR) were monitored weekly. Blood samples were analyzed for hematological and immunological parameters, while intestinal histology was assessed. Results indicated that feed intake was significantly reduced in the highest supplementation (LSE3) group (*P* < 0.0001), while weight gain and FCR improved significantly compared to the control (*P* < 0.0001). Although antibody titers against Newcastle Disease and Infectious Bronchitis increased, differences were not statistically significant. Histomorphometric analysis revealed significantly longer villus height and decrease in crypt depth (*P* = 0.001) in LSE3. Overall, *Lactobacillus* and serratopeptidase supplementation (LSE3) improved growth performance and intestinal morphology, with the highest inclusion level yielding the best results, suggesting potential benefits for broiler production efficiency.

## Introduction

Poultry production is a vital sector of the livestock industry, supplying a significant proportion of global animal protein. However, the increasing demand for antibiotic-free poultry products and the challenges posed by gut health disorders, immune suppression, and poor nutrient absorption necessitate alternative strategies to improve broiler performance (Ajmal et al., 2023; Khan et al., 2022; Mahmood et al., 2024). Among the potential alternatives, serratiopeptidase (SP), a proteolytic enzyme, and *Lactobacillus*, a well-documented probiotic, have gained attention for their beneficial effects on broiler health, immunity, and intestinal function (Boonmee et al., 2024; Khan and Naz, 2013; Nguyen et al., 2024; Sultan et al., 2024). Serratiopeptidase and *Lactobacillus* support gut health through complementary mechanisms. Serratiopeptidase, as a proteolytic enzyme, helps degrade inflammatory proteins and biofilms, reducing intestinal inflammation and enhancing nutrient absorption (Nair, 2022). *Lactobacillus*, a probiotic, improves gut health by balancing the intestinal microflora, inhibiting pathogenic bacteria, producing beneficial metabolites (e.g., lactic acid), and strengthening the gut barrier function (Chen et al., 2024). Together, these supplements promote a healthier intestinal environment, better immunity, and improved nutrient utilization in broilers. In modern poultry farming, birds are frequently exposed to various stressors, including heat stress, infections, and environmental changes, which compromise their immune system and growth performance (Khan et al., 2023). Additionally, intestinal health plays a pivotal role in optimizing nutrient absorption, maintaining feed efficiency, and preventing gut-associated infections (Sultan et al., 2024). Thus, enhancing gut microbiota balance, immune responses, and digestive efficiency is essential for sustainable broiler production (Uzair et al., 2025). The ban on antibiotics as growth promoters (AGPs) in poultry due to antimicrobial resistance (AMR) concerns has led to the search for alternative feed additives.

Probiotics are among the most promising candidates, as they enhance nutrient digestion, gut microbiota stability, and immune function while reducing dependence on AGPs (Susalam et al., 2024). Serratiopeptidase (SP) is a proteolytic enzyme derived from *Serratia marcescens* that has been widely used in human medicine for its anti-inflammatory, fibrinolytic, and mucolytic properties (Nair et al., 2022). Studies suggest that SP may improve gut integrity, enhance protein digestibility, and reduce inflammatory stress in poultry, leading to better growth performance and feed efficiency (Mushtaq et al., 2024). Probiotics, particularly *Lactobacillus* spp., play a crucial role in maintaining gut homeostasis by modulating microbial populations, improving gut morphology, and boosting immune responses (Chen et al., 2024; Wickramasuriya et al., 2022). Therefore, we hypothesize that the combined supplementation of serratiopeptidase and *Lactobacillus* will synergistically improve growth performance, immune function, hematological profiles, and intestinal histomorphology in broiler chickens. The synergistic combination of *Lactobacillus* and serropeptidase may offer a dual approach to enhancing digestive function, reducing gut inflammation, and improving nutrient bioavailability, thereby benefiting overall broiler health and productivity. This study aims to evaluate the effects of serratiopeptidase and *Lactobacillus* supplementation on growth performance, immune responses, hematological parameters and intestinal histomorphology in broilers.

## Materials and methods

### Experimental design and management practices

A total of 320-day-old male broiler chicks (Hubbard) were randomly assigned to four treatment groups, with each group consisting of five replications of 16 chicks. The birds were housed in an open-sided poultry facility, ensuring adequate ventilation.

During the brooding phase, all chicks were housed in an open-sided house equipped with individual floor pens. Each pen measured 1.2 *m* × 1.5 m and was constructed using wire mesh and wooden partitions to prevent bird mixing while allowing adequate ventilation. The floor of each pen was covered with a 5 cm deep layer of clean, dry wood shavings as litter material, which was regularly stirred and replaced as needed to maintain hygiene. A temperature-controlled environment was ensured during brooding, using an incandescent lamp as the primary heat source. The brooding temperature was maintained at 32–35°C during the first few days and was gradually reduced to 22–24°C by the end of the brooding period, in accordance with standard poultry management guidelines. Fresh water and feed were provided ad libitum, and all pens were uniformly equipped to ensure similar management conditions across treatments.

The birds were divided into the following four dietary treatments**:** Control**:** Standard basal diet without supplementation (Table 1); Diet supplemented with Lactobacillus at 2 × 10¹⁰ CFU/kg **+** 5 mg/kg SP (LSE1); Diet supplemented with Lactobacillus at 3 × 10¹⁰ CFU/kg **+** 5 mg/kg SP (LSE2); Diet supplemented with Lactobacillus at 4 × 10¹⁰ CFU/kg **+** 5 mg/kg SP (LSE3).

**Table 1:** Ingredients and chemical composition of basal feed.

Ingredients	Starter Phase (1-21 days)	Finisher phase (22-42 days)
Yellow corn	53.21	60.75
Soybean meal (48 % CP)	37.92	25.00
Corn gluten meal (34 % CP)	2.00	7.10
Corn oil	2.20	2.80
Dicalcium phosphate	2.30	2.05
Limestone	0.83	0.68
NaCl	0.45	0.50
Vitamin and minerals premix^1^	0.50	0.50
DL-Methionine	0.20	0.10
L-Lysine HCl	0.22	0.37
L-Threonine	0.11	0.10
Choline chloride	0.05	0.05
Chemical composition		
ME, kcal/kg	3000	3150
Crude protein, %	22.5	21.30
Crude fat, %	04.5	05.25
Methionine, %	0.55	0.44
Lysine, %	1.42	1.23
Sulfur amino acids, %	0.96	0.80
Threonine, %	0.95	0.85
Calcium, %	1.05	0.90
Available phosphorus, %	0.50	0.45

1 Provided per kg of diet: vitamain A, 8000 IU; vitamain D3,2000 IU; vitamain E,15 IU; vitamain K3, 1.5 mg; thiamine, 2 mg; riboflavin, 5 mg; pyridoxine, 5 mg; vitamain B12, 0.02 mg; folic acid, 0.7 mg; nicotinic acid, 40 mg; pantothenic acid, 12 mg; biotin, 0.2 mg b Provided per kg of diet: copper, 10 mg (as copper sulfate); iron, 90 mg (as ferrous sulfate); manganese, 100 mg (as manganese sulfate); zinc, 100 mg (as zinc sulfate); selenium, 0.3 mg (as sodium selenite); iodine, 0.5 mg (as calcium iodate).

Lactobacillus was obtained from CLOSTAT® (Kemin Industries, Inc., Des Moines, IA, USA). Serratopeptidase was purchased from Takeda Chemical Industries, Japan.

The experiment lasted six weeks. Birds were maintained on a continuous lighting schedule for the first 24 hours, followed by 23 hours of light and 1 hour of darkness per day throughout the study. A standard commercial broiler diet was provided *ad libitum*, ensuring optimal nutrient availability. Fresh, clean drinking water was available ad libitum throughout the experiment, supplied via an automatic watering system. Standard management practices were followed to ensure the well-being and optimal performance of the birds under experimental conditions.

### Growth parameters

Daily feed consumption was recorded by measuring the total amount of feed offered and subtracting the leftover feed at the end of each day. The overall feed intake was calculated at the end of the trial. The birds were weighed weekly to assess body weight gain. This was determined by subtracting the initial body weight from the final body weight at the end of each week. The feed conversion ratio was calculated weekly for each treatment group by dividing the total amount of feed consumed by the total body weight gain. This parameter was used to evaluate feed efficiency across different dietary treatments.

### Blood collection and analysis

At the conclusion of the experiment (42 day), blood samples were collected from two birds per replicate group using sterile syringes via the wing vein. A total of 3 mL of blood was drawn from both control and treated birds and divided into two separate tubes: one containing EDTA for hematological and immunological assessments and another without EDTA for serum separation. Serum was obtained by centrifuging the samples at 15,000 rpm for 10 minutes. The collected serum was then analyzed for antibodies specific to Infectious Bronchitis (IB) and Newcastle Disease (ND) through enzyme-linked immunosorbent assay (ELISA), hemagglutination (HA), and hemagglutination inhibition (HI) tests.

### Hematological parameters

Hematological assessments included erythrocyte count, Packed Cell Volume (PCV), hemoglobin concentration, White Blood Cell (WBC) count, and differential leukocyte count (DLC) as markers of immune response. Hemoglobin levels were assessed using the Van Sahli method, while packed cell volume (PCV) was determined with a Hacksley hematocrit centrifuge (UK). Total and differential white blood cell counts were performed using a Neubauer hemocytometer, in accordance with procedures adapted from Kalsoom et al. (n.d.). Mean corpuscular hemoglobin (MCH) was calculated based on leukocyte differential counts. The erythrocyte sedimentation rate (ESR) was measured using the Westergren technique, as outlined by Asghar et al. (n.d.).

### Ileum histology

Following slaughter (42 days), ileal samples from each bird were fixed in 10 % formalin and later processed using paraffin embedding. Cross-sections of 5 µm thickness were cut and stained with hematoxylin and eosin (H&E) for histomorphometric evaluation. Imaging and measurements were conducted using an Olympus light microscope paired with ProgRes CapturePro software (version 2.7; Jenoptik, Jena, Germany). For each sample, only structurally intact and clearly defined villi—ten in total—were selected for analysis. Villus height was measured from the apex to the junction with the crypt, and crypt depth was assessed from the base to the opening of the crypt. The average villus height and crypt depth were computed for each sample, and the villus height-to-crypt depth ratio was also determined.

### Statistical analysis

The data were analyzed using a Completely Randomized Design (CRD). All results were expressed as mean ± standard error (SE). A one-way analysis of variance (ANOVA) was performed using SPSS software (version 21) to determine significant differences among treatment groups. Mean comparisons were conducted using the Tukey HSD test at a significance level of *P* < 0.05. The mathematical model used was as follows:

Yᵢⱼ = μ + τᵢ + εᵢⱼ

Where:

*Yᵢⱼ* = Observation for the *jᵗʰ* replicate of the *iᵗʰ* treatment

*μ* = Overall mean

*τᵢ* = Effect of the *iᵗʰ* treatment

*εᵢⱼ* = Random error associated with each observation

## Results

The effect of *Lactobacillus* and serratopeptidase enzyme supplementation on the feed intake of broiler chicks is presented in Table 2. Feed intake during the first week did not differ significantly among the groups (*P* = 0.43). However, from the second week onward, significant differences were observed among the treatments (*P* < 0.05). The control group had the highest feed intake across all weeks, while LSE3 exhibited the lowest values.

**Table 2:** Effect of *Lactobacillus* and serropeptidase Enzymes on feed intake of broiler chicks.

Group	Weekly feed intake of broiler					Total Feed Intake
	week-I	week-II	week-III	week-IV	week-V	
	Means ± SE	Means ± SE	Means ± SE	Means ± SE	Means ± SE	Means ± SE
Control	136 ± 2.55	365.77^a^ ± 2.68	615.3^a^ ± 2.88	896.33^a^ ± 4.33	1194.7^a^ ± 6.88	3206^a^ ± 5.51
LSE1	137.2 ± 2.43	367.43^a^ ± 3.26	614.67^a^ ± 4.45	884.3^b^ ± 3.02	1192.3^a^ ± 7.45	3196^b^ ± 4.72
LSE2	135.1 ± 3.51	365.21^a^ ± 5.62	597.3^b^ ± 6.45	874.3^c^ ± 2.75	1162.3^b^ ± 8.46	3128^c^ ± 7.43
LSE3	134.3 ± 3.81	355.33^b^ ± 6.28	586.0^c^ ± 5.15	833.3^d^ ± 5.45	1156.2^b^ ± 4.33	3062^d^ ± 5.75
P-value	0.43	0.002	0.001	0.0002	0.001	0.001

Mean in column having different superscripts are significantly different at α = 0.05.

LSE: *Lactobacillus* and serropeptidase Enzyme.

LSE1; *Lactobacillus* at 2 × 10¹°CFU/kg + 5 mg/kg serropeptidase; LSE2: 3 × 10¹°CFU/kg + 5 mg/kg serropeptidase; LSE3: 4 × 10¹°CFU/kg + 5 mg/kg serropeptidase.

By the second week, LSE3 showed a significantly lower feed intake (356.3 ± 2.18 g) compared to the control (366.67 ± 0.88 g; *P* = 0.002). This trend continued into the third week, where feed intake was significantly reduced in LSE3 (588.0 ± 1.15 g) compared to the control (616.3 ± 0.88 g; *P* < 0.0001). The reduction in feed intake was more pronounced in the fourth and fifth weeks, with LSE3 recording the lowest values (830.3 ± 1.45 g and 1155.2 ± 2.33 g, respectively), significantly differing from the control group (894.33 ± 2.33 g and 1193.7 ± 0.88 g, respectively; *P* < 0.0001).

Total feed intake over the entire study period followed a similar pattern, with the control group showing the highest cumulative feed intake (3208 ± 2.5 g), while LSE3 had the lowest (3060 ± 5.7 g; *P* < 0.0001).

The effect of *Lactobacillus* and serratopeptidase enzyme supplementation on the weight gain of broiler chicks is presented in Table 3. Weight gain during the first week was not significantly different among the groups (*P* = 0.06). However, from the second week onward, significant differences were observed (*P* < 0.05). The LSE3 group exhibited the highest weight gain across all weeks, while the control group had the lowest values.

**Table 3:** Effect of *Lactobacillus* and serropeptidase Enzymes on weight gain of broiler chicks.

Group	Weekly weight gain of broiler					Total Weight Gain
	week-I	week-II	week-III	week-IV	week-V	
	Means ± SE	Means ± SE	Means ± SE	Means ± SE	Means ± SE	Means ± SE
Control	116.5 ± 4.75	136.0^d^ ± 1.63	376.0^d^ ± 2.73	485.1^c^ ± 2.83	616.45^c^ ± 3.41	1725.3^d^ ± 4.46
LSE1	121.4 ± 3.45	137.3^c^ ± 0.78	394.0^c^ ± 2.15	486.4^c^ ± 2.66	632.11^a^ ± 3.52	1773.0^c^ ± 2.34
LSE2	127.1 ± 5.72	145.3^b^ ± 1.40	406.0^b^ ± 2.52	512.2^b^ ± 2.55	623.22^b^ ± 3.23	1808.7^b^ ± 4.63
LSE3	128.6 ± 7.61	153.6^a^ ± 1.30	417.6^a^ ± 2.45	523.6^a^ ± 2.20	624.71^b^ ± 5.12	1845.0^a^ ± 6.62
P-value	0.06	0.001	0.002	0.0004	0.0001	0.0001

Mean in column having different superscripts are significantly different at α = 0.05.

LSE: *Lactobacillus* and serropeptidase Enzyme.

LSE1; *Lactobacillus* at 2 × 10¹°CFU/kg + 5 mg/kg serropeptidase; LSE2: 3 × 10¹°CFU/kg + 5 mg/kg serropeptidase; LSE3: 4 × 10¹°CFU/kg + 5 mg/kg serropeptidase.

By the second week, LSE3 demonstrated a significantly higher weight gain (152.6 ± 1.20 g) compared to the control (132.0 ± 1.73 g; *P* < 0.0001). A similar trend was observed in the third week, with LSE3 showing the highest weight gain (414.6 ± 1.45 g) compared to the control (375.0 ± 1.73 g; *P* = 0.0002). This pattern continued in the fourth and fifth weeks, where LSE3 recorded significantly greater weight gains (521.3 ± 1.20 g and 624.6 ± 2.0 g, respectively) compared to the control group (485.0 ± 1.73 g and 613.67 ± 1.76 g, respectively; *P* < 0.0001).

Total weight gain over the entire study period followed the same trend, with LSE3 achieving the highest cumulative weight gain (1843.0 ± 4.0 g), significantly higher than the control (1723.3 ± 3.3 g; *P* = 0.0001).

The effect of *Lactobacillus* and serratopeptidase enzyme supplementation on the feed conversion ratio (FCR) of broiler chicks is presented in Table 4. During the first week, no significant differences in FCR were observed among groups (*P* = 0.07). However, from the second week onward, significant improvements in FCR were recorded in birds supplemented with *Lactobacillus* and serratopeptidase enzymes (*P* < 0.05).

**Table 4:** Effect of *Lactobacillus* and serropeptidase Enzymes on FCR of broiler chicks.

Group	Weekly FCR of broiler					Total FCR
	week-I	week-II	week-III	week-IV	week-V	
	Means ± SE	Means ± SE	Means ± SE	Means ± SE	Means ± SE	Means ± SE
Control	1.15 ± 0.01	1.76^a^ ± 0.03	1.65^a^ ± 0.02	1.85^a^ ± 0.01	1.95^a^ ± 0.03	1.87^a^ ± 0.01
LSE1	1.11 ± 0.02	1.67^b^ ± 1.0	1.56^b^ ± 0.03	1.82^b^ ± 0.02	1.84^b^ ± 0.01	1.78^b^ ± 0.03
LSE2	1.04 ± 0.02	1.54^c^ ± 0.01	1.45^c^ ± 0.03	1.73^c^ ± 0.03	1.85^b^ ± 0.04	1.74^c^ ± 0.01
LSE3	1.01 ± 0.05	1.34^d^ ± 0.01	1.42^d^ ± 0.01	1.59^d^ ± 0.02	1.84^c^ ± 0.03	1.66^d^ ± 0.02
P-value	0.07	0.0000	0.0003	0.0000	0.0001	0.0000

Mean in column having different superscripts are significantly different at α = 0.05.

LSE: *Lactobacillus* and serropeptidase Enzyme.

LSE1; *Lactobacillus* at 2 × 10¹°CFU/kg + 5 mg/kg serropeptidase; LSE2: 3 × 10¹°CFU/kg + 5 mg/kg serropeptidase; LSE3: 4 × 10¹°CFU/kg + 5 mg/kg serropeptidase.

By the second week, the LSE3 group had the lowest FCR (2.33 ± 0.01), significantly lower than the control group (2.77 ± 0.03; *P* < 0.0001). A similar pattern was observed in the third and fourth weeks, where LSE3 maintained the lowest FCR (1.41 ± 0.01 and 1.59 ± 0.02, respectively), significantly better than the control (1.64 ± 0.02 and 1.84 ± 0.01; *P* < 0.0003). In the fifth week, LSE3 again demonstrated the best FCR (1.83 ± 0.03), significantly lower than the control group (1.94 ± 0.03; *P* < 0.0001).

Cumulative FCR over the entire study period followed the same trend, with LSE3 achieving the most efficient feed conversion (1.65 ± 0.02), significantly better than the control (1.86 ± 0.01; *P* < 0.0001). These findings indicate that supplementation with *Lactobacillus* and serratopeptidase enzymes effectively enhanced feed efficiency, with the LSE3 group showing the most pronounced improvements. The effect of *Lactobacillus* and serratopeptidase enzyme supplementation on the immune response of broiler chicks is presented in Table 5. The antibody titers against Newcastle Disease (ND) and Infectious Bronchitis (IB) showed an increasing trend with enzyme supplementation. However, the differences among groups were not statistically significant for either ND (*P* = 0.0870) or IB (*P* = 0.0595).

**Table 5:** Effect of *Lactobacillus* and serropeptidase Enzymes on immunity of broilers chicks.

Groups	Means ± SE (Antibody Titer)	
	ND	IB
Control	4.12 ± 0.11	4012 ± 12.03
LSE1	4.45 ± 0.28	4023 ± 31.30
LSE2	4.54 ± 0.43	4042 ± 23.28
LSE3	4.77 ± 0.67	4056 ± 21.42
P-value	0.0560	0.0595

LSE: *Lactobacillus* and serropeptidase Enzyme.

LSE1; *Lactobacillus* at 2 × 10¹°CFU/kg + 5 mg/kg serropeptidase; LSE2: 3 × 10¹°CFU/kg + 5 mg/kg serropeptidase; LSE3: 4 × 10¹°CFU/kg + 5 mg/kg serropeptidase.

Despite the lack of statistical significance, birds in the LSE3 group exhibited the highest antibody titers for both ND (4.66 ± 0.57) and IB (4066 ± 0.42), compared to the control group (4.00 ± 0.01 for ND and 4002 ± 0.03 for IB).

The effect of *Lactobacillus* and serratopeptidase enzyme supplementation on the hematological parameters of broiler chicks is summarized in Table 6. No statistically significant differences were observed for erythrocyte count (*P* = 0.3914), hemoglobin concentration (*P* = 0.9012), hematocrit percentage (*P* = 0.1605), or leukocyte count (*P* = 0.4925).

**Table 6:** Effect of *Lactobacillus* and serropeptidase Enzymes on haematological aspects of broilers chicks.

Group	Hematological aspects of broiler			
	Erythrocytes (10^6^/mm^3^)	Hemoglobin (g/100 mL)	Hematocrit (%)	Leukocyte (10^3^/mm^3^)
	Means ± SE	Means ± SE	Means ± SE	Means ± SE
Control	2.44 ± 1.06	8.55 ± 1.03	27.44 ± 1.06	18.41 ± 1.13
LSE1	2.51 ± 1.06	8.61 ± 1.02	27.91 ± 1.08	18.92 ± 1.18
LSE2	2.54 ± 1.09	8.71 ± 1.04	28.16 ± 1.05	19.82 ± 1.28
LSE3	2.61 ± 1.09	8.85 ± 1.04	28.51 ± 1.07	20.86 ± 1.24
P-value	0.3914	0.9012	0.1605	0.4925

LSE: *Lactobacillus* and serropeptidase Enzyme.

LSE1; *Lactobacillus* at 2 × 10¹°CFU/kg + 5 mg/kg serropeptidase; LSE2: 3 × 10¹°CFU/kg + 5 mg/kg serropeptidase; LSE3: 4 × 10¹°CFU/kg + 5 mg/kg serropeptidase.

Among the treatment groups, the highest erythrocyte count (2.62 ± 0.09 × 10⁶/mm³), hemoglobin concentration (8.84 ± 0.04 g/100 mL), hematocrit (28.50 ± 0.07 %), and leukocyte count (20.87 ± 0.24 × 10³/mm³) were recorded in the LSE3 group, while the lowest values were observed in the control group.

The effect of *Lactobacillus* and serratopeptidase enzyme supplementation on intestinal histomorphological parameters of broiler chicks is presented in Table 7. Significant improvements (*P* = 0.001) were observed in villus height, villus width, and crypt depth among the treatment groups compared to the control. The highest villus height (1372.07 ± 9.01 mm) and villus width (286.18 ± 10.3 mm) were recorded in the LSE3 group, followed by LSE2, LSE1, and the control, indicating a dose-dependent effect of supplementation. Conversely, crypt depth was significantly reduced in the supplemented groups, with LSE3 showing the lowest depth (139.32 ± 8.07 mm) compared to the control (146.34 ± 4.8 mm). A lower crypt depth is generally associated with improved nutrient absorption and intestinal health.

**Table 7:** Effect of *Lactobacillus* and serropeptidase Enzymes on Intestinal histomorphological changes of broilers chicks.

Group	Villus Height (mm)	Villus Width (mm)	Crypt Depth (mm)
Control	1286.44^d^ ± 13.1	275.65 ± 3.1 ^d^	145.33 ± 4.4 ^a^
LSE1	1304.11^c^ ± 17.1	281.55 ± 9.3^c^	145.24 ± 8.2^b^
LSE2	1359.20^b^ ± 4.7	284.55 ± 9.3 ^b^	141.21 ± 4.6 ^c^
LSE3	1373.17^a^ ± 7.01	285.132 ± 10.3 ^a^	138.33 ± 8.7 ^d^
P Value	0.001	0.001	0.001

Mean in column having different superscripts are significantly different at α = 0.05.

LSE: *Lactobacillus* and serropeptidase Enzyme.

LSE1; *Lactobacillus* at 2 × 10¹°CFU/kg + 5 mg/kg serropeptidase; LSE2: 3 × 10¹°CFU/kg + 5 mg/kg serropeptidase; LSE3: 4 × 10¹°CFU/kg + 5 mg/kg serropeptidase.

## Discussion

Feed additives have long been used in poultry nutrition. Numerous experimental studies have highlighted the importance of probiotics and proteolytic enzymes as natural growth promoters in broiler production. In the current study, the results indicate distinct changes in performance parameters. The combined supplementation of Lactobacillus and serratiopeptidase at varying inclusion levels showed positive effects on broiler performance, notably in promoting body weight gain and feed conversion ratio. Among the treatment groups, LSE3 exhibited the greatest improvements, with the highest body weight, and a notably enhanced feed conversion ratio (FCR), accompanied by a significant decrease in feed intake. These enhancements in growth performance may be linked to the modulation of ghrelin gene expression at different regions of the gastrointestinal tract (Okenvi et al., 2023). Specifically, increased expression of ghrelin in the proventriculus and its reduced expression in the intestinal segment have been associated with improved nutrient assimilation (Abdalla, 2015). Probiotic and enzyme supplementation can influence ghrelin activity both within the gut and the central nervous system (CNS), thereby affecting metabolic outcomes (Jazi et al., 2017). Additionally, the alteration of intestinal gene expression by probiotics and serratiopeptidase may contribute to enhanced nutrient uptake by stimulating enterocyte proliferation (hyperplasia). This cellular activity, along with the enlargement and increased number of goblet cells, may extend the length and surface area of the intestinal villi, thereby improving the absorptive efficiency of the intestine. This enhanced absorption contributes to greater body weight gain, ultimately improving overall production performance parameters (Jazi et al., 2017).

The lower feed intake in LSE3, especially from the second week onward, suggests improved digestive efficiency due to enhanced enzymatic activity and beneficial microbial colonization, which contribute to better nutrient absorption (Mushtaq et al., 2024). The significant improvement in FCR in the LSE3 group supports previous findings that probiotics and exogenous enzymes enhance feed efficiency by improving digestion and reducing feed wastage (Sultan et al., 2024; Yu et al., 2024). The lowest FCR in LSE3 suggests that *Lactobacillus* and serratiopeptidase supplementation enhanced digestive enzyme activity, reducing undigested feed residues and optimizing metabolic energy utilization. The consistent reduction in FCR across all weeks suggests that the synergistic action of probiotics and enzymes maintained efficiency throughout the growth period. Overall, the findings indicate that *Lactobacillus* and serratiopeptidase supplementation improved nutrient utilization, reduced feed intake, enhanced weight gain, and significantly improved feed efficiency, making them a promising alternative to conventional growth promoters.

The observed increase in antibody titers against ND and IB in broilers supplemented with *Lactobacillus* and serratopeptidase, although not statistically significant, suggests a potential immunomodulatory effect of these additives. Probiotic bacteria, particularly *Lactobacillus* spp., are known to enhance gut-associated lymphoid tissue (GALT) activity, leading to improved systemic immune responses through modulation of cytokine expression and increased immunoglobulin production (Alqahtani et al., 2024; Mirnawati et al., 2024; Rashid et al., 2023). The slight elevation in antibody titers in the LSE3 group aligns with previous studies demonstrating that probiotic supplementation can enhance vaccine-induced humoral immunity in poultry (Alqahtani et al., 2024; Jazi et al., 2017; Mehmood et al., 2023).

Serratopeptidase, a proteolytic enzyme, may further contribute to immune enhancement by reducing intestinal inflammation and improving nutrient absorption, indirectly supporting immune function (Mushtaq et al., 2024). Studies have reported that proteolytic enzymes can facilitate antigen presentation and lymphocyte proliferation, thereby strengthening vaccine responses (Chapman et al., 2006). Overall, while the results do not provide definitive evidence of enhanced immunity, they suggest that *Lactobacillus* and serratopeptidase supplementation may contribute to a more robust immune profile in broilers, warranting further investigation under different vaccination protocols or stress conditions.

The use of varying levels of probiotics and serratiopeptidase resulted in a significant improvement in gut morphology. The LSE3 treatment group exhibited notably greater villus height and width, along with reduced crypt depth, compared to the other treatment groups. Numerous studies have suggested that the growth-promoting effects of probiotics in poultry are primarily due to improvements in the gastrointestinal ecosystem. These enhancements lead to a healthier intestinal environment, better integrity of the mucosal barrier, stimulation of mucosal proliferation, and improved digestive and immune functions—ultimately contributing to overall broiler health (Dabool et al., 2024; Yu et al., 2024). The observed improvements in intestinal histomorphology following *Lactobacillus* and serratopeptidase supplementation indicate enhanced gut health and nutrient absorption efficiency. Probiotic supplementation, particularly with *Lactobacillus* species, has been widely reported to promote intestinal development by enhancing villus height and width while reducing crypt depth, which aligns with the findings of this study (Human et al., 2019). The increase in villus height and width in the LSE3 group suggests a greater surface area for nutrient absorption, leading to improved growth performance. The concurrent reduction in crypt depth indicates lower epithelial turnover, which is associated with improved gut efficiency and reduced energy expenditure on intestinal cell renewal. *Lactobacillus* plays a crucial role in maintaining gut homeostasis by modulating the gut microbiota, enhancing the gut barrier function, and exerting anti-inflammatory effects (Ashayerizadeh et al., 2016). The ability of lactic acid bacteria to modify the gut environment likely contributed to the observed changes in villus architecture, as previously reported by Mushtaq et al. (2024). Furthermore, *Lactobacillus* supplementation has been shown to inhibit the colonization of pathogenic bacteria, including *Escherichia coli*, which may otherwise compromise gut integrity (Yu et al., 2015). The synergistic action of *Lactobacillus* and serratiopeptidase may have further enhanced gut health by modulating inflammatory responses and promoting epithelial repair. The histological examination of the duodenum in this study supports the hypothesis that probiotics improve intestinal integrity. Mushtaq et al. (2024) demonstrated that *B. subtilus* and serropeptidase supplementation significantly altered villus morphology, leading to better nutrient absorption and feed efficiency. Similarly, in the present study, the positive effects of *Lactobacillus* and serratiopeptidase supplementation on villus height and width, along with reduced crypt depth, confirm their role in improving intestinal architecture. These findings support previous reports that probiotic supplementation contributes to enhanced gut development and overall broiler performance.

## Conclusion

Supplementation with *Lactobacillus* and serratopeptidase enzyme improved broiler performance by reducing feed intake while enhancing weight gain, feed conversion efficiency, and intestinal morphology, with the LSE3 group showing the most pronounced benefits.

## Disclosure statement

No potential conflict of interest was reported by author(s)

## Ethical statement

The Committee on Animal Rights and Welfare, The University of Agriculture, Peshawar, Pakistan approved this study (FAHVS/122/2023).

### Data availability

Data will be made available from the authors upon reasonable request.

## Declaration

English was corrected for readability through AI (chatgpt) which was reviewed for correction.

## Declaration of competing interest

Authors declare no competing interest
